# Orthopaedic Trauma Theatre Efficiency in the COVID-19 Pandemic: Are We Returning to Normality?

**DOI:** 10.7759/cureus.13221

**Published:** 2021-02-08

**Authors:** Faizan Arshad, Umar-Khetaab Hanif, Arslan Arshad, Muhammad I Chaudary, Amir Khan, Joshua Kelleher, Salman Sadiq, Abdus Samee Wasim, Fouad Chaudhry

**Affiliations:** 1 Trauma and Orthopaedics, Russells Hall Hospital, Dudley, GBR; 2 Emergency Department, Hillingdon Hospital, Uxbridge, GBR; 3 Trauma and Orthopaedics, Queen Elizabeth Hospital, Birmingham, GBR

**Keywords:** trauma, theatre, efficiency, covid-19, pandemic

## Abstract

Background

Recent studies have shown a decline in theatre efficiency and productivity coinciding with the coronavirus disease 2019 (COVID-19) pandemic. In this study, we evaluate trauma theatre task efficiency in three different time periods (April 2019, April 2020, and November 2020), and analyse if productivity has altered since the start of the pandemic.

Methods

The records of a total of 320 patients who underwent orthopaedic trauma surgery at a large district general hospital in April 2019, April 2020 (during the first wave of the pandemic) and November 2020 (during the second wave of the pandemic) were analysed. Primary outcomes measured include time to get to the theatre, anaesthetic preparation time, the sum of time of anaesthesia and surgical preparation time, duration of surgery and time to transfer to recovery. Patient demographics as well as the type of surgery were also analysed.

Results

The time to get to the theatre and anaesthetic preparation time significantly increased in April 2020 (p<0.05) but fell in November 2020 with no significant difference in comparison to before the pandemic in April 2019 (p>0.05). The duration of surgery and time to transfer to recovery significantly increased in April 2020 (p<0.05) and though reduced in November 2020, was still significantly greater in comparison to April 2019 (p<0.05). In April 2020, the proportion of patients aged 18-65 was just 26% as compared to 35% in April 2019. This figure rose again to 45% in November 2020. The number of hip fracture procedures remained similar during the three time periods, with 32, 32 and 36 hip fracture operations in April 2019, April 2020 and November 2020, respectively.

Conclusion

While operating theatres' efficiency decreased during the first wave of the COVID-19 pandemic, it increased again in the second wave, coming close to the ‘normal’ levels before the pandemic struck.

## Introduction

The coronavirus disease 2019 (COVID-19) pandemic has posed an unprecedented challenge to modern healthcare services. Before the pandemic, operating theatres were striving to achieve high efficiency and productivity [1-4]. However, the pandemic has brought a paradigm shift of priorities; all members of the theatre team are now guided to focus on patient and staff safety in measures to prevent the transmission of the COVID-19 virus [5-6]. With efforts focused on preventing the virus’ transmission, efficiency and productivity seem to have been affected by this.

Several recent studies have shown a fall in operating room efficiency when the pandemic initially affected healthcare systems across the world [7-9]. However, at the time of writing, there are no studies that have analysed how the efficiency of operating theatres has changed over the many months since the onset of the pandemic in March 2020 [10].

The United Kingdom (UK) government announced a national ‘lockdown’ in April 2020 [11] during the ‘first wave’ of the pandemic and then again in November 2020 [12] during the ‘second wave’. During the national lockdown, the whole population in the country was instructed to stay at home except for exceptional circumstances [11-12]. Both these time periods represent peaks of the rate of spread of the COVID-19 virus and thus are useful time periods to assess its impact on healthcare services. In this article, we explore the operating patterns and the operating theatre efficiency for orthopaedic trauma theatres during three time periods: before the pandemic in April 2019, at the beginning of it during the ‘first wave’ in April 2020 and during the ‘second wave’ in November 2020.

## Materials and methods

All patients who underwent surgery in orthopaedic trauma theatres from the beginning to the end of April 2019, April 2020 and November 2020 were extracted from the trust’s emergency theatres database at Russells Hall Hospital, a large district general hospital in the UK. An illustrative summary of the patients analysed is detailed in Table 1. For each patient in the three cohorts, the date and time of surgery, the type of the surgical procedure and a range of outcomes were extracted. The primary outcomes measured include time to get to the theatre, anaesthetic preparation time, the sum of time of anaesthesia and surgical preparation time, duration of surgery and time to transfer to recovery. A detailed description of these outcomes is displayed in Table 2. Where any of these parameters seemed to be erroneously recorded, for example, if the time of surgery was recorded as after the time of transfer to recovery, the whole dataset was excluded. The whole database of extracted patients was assessed by two independent reviewers (F.A. and U.H.) and any difference of opinion was resolved by the senior author (F.C.).

**Table 1:** Identification of patients included in the analysis *Other cancellation reasons included patients being unfit for surgery, awaiting investigations or transfer to another operating list

	April 2019	April 2020	November 2020
Total patients extracted from the database for the whole month	165	109	174
Duplicate entries deleted	16	10	30
Cases cancelled* (cases cancelled due to shortage of time in brackets)	25 (7)	19 (10)	23 (7)
Erroneous times entered due to human error	2	2	1
Sample size analysed (n)	122	78	120

**Table 2:** Description of primary outcomes

Outcome	Description
Time to get to the theatre	Time between the patient being called to the theatre to their entry in the operating suite. This typically involves a member of the staff travelling to the ward, collecting the patient and transporting them to the anaesthetic or operation room, where they would be anaesthetised prior to the surgical procedure.
Anaesthetic preparation time	Time between the patient’s entry into the operating suite and the initiation of anaesthesia.
Time of anaesthesia + surgical preparation time	Time between the induction of anaesthesia and the initiation of the surgical procedure. This includes the total time taken for the anaesthesia and the surgical preparation such as positioning the patient onto the operating table.
Duration of surgery	The total duration of surgery, from its initiation to its completion.
Time to transfer to recovery	Time between the completion of surgery and patient leaving the operating suite for recovery. This time may include the extubation of the patient if it was chosen to be performed inside the operating theatre.

The three cohorts of patients were divided into ‘hip fracture operations’ and all ‘other operations’; hip fracture operations included all surgical procedures for the treatment of neck of femur fractures including dynamic hip screw fixation, trochanteric femoral nailing, hip hemiarthroplasty and total hip arthroplasty.

All the data were imported in Microsoft Excel (Microsoft Corporation, Redmond, WA) and analysed. Ninety-five per cent (95%) confidence intervals were plotted on the chart to visually represent statistical differences. The t-test was used to determine statistical significance with a significance level set at 0.05.

## Results

Operating patterns

**Figure 1:**
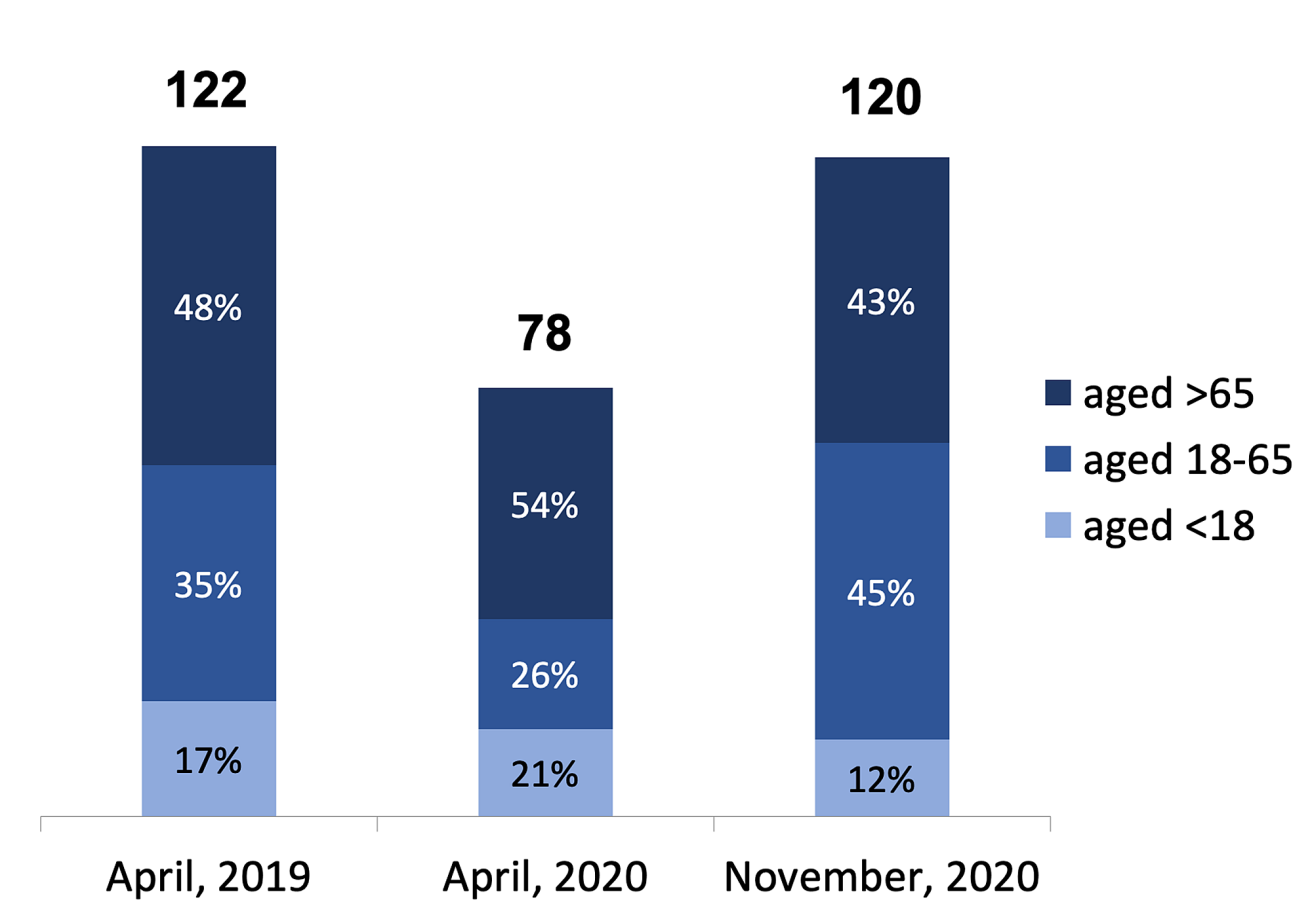
Age groups of patients undergoing orthopaedic trauma surgery

A total of 122 patients underwent surgery in orthopaedic trauma theatres in the month of April 2019, 78 in April 2020 and 120 in November 2020. In April 2020, during the first wave of the COVID-19 pandemic, 54% of operations were performed on patients over 65 years of age, whereas 48% and 43% of operations were performed on this age group in April 2019 and November 2020, respectively. In April 2020, only 26% of patients operated on were aged between 18 and 65 as compared to 35% and 45% during April 2019 and November 2020, respectively. In April 2019 and November 2020, this figure was much higher at 35% and 45%, respectively. Figure 1 illustrates the different age groups who underwent procedures in orthopaedic trauma theatres.

**Figure 2:**
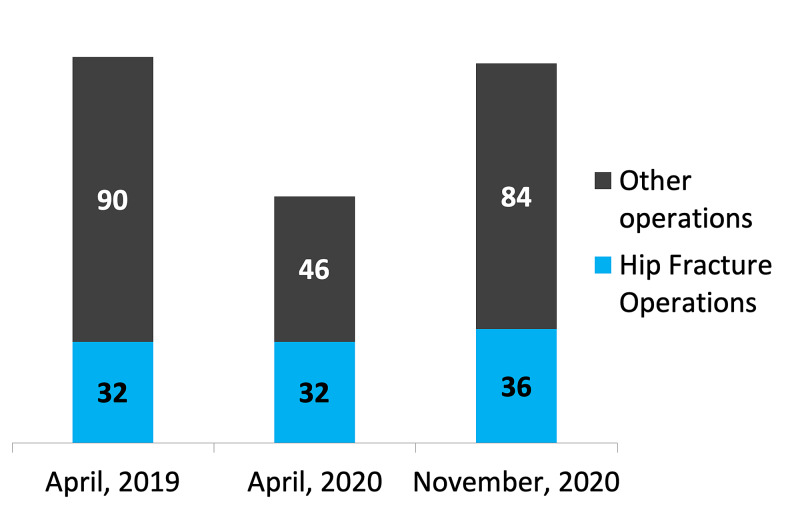
Types of operations performed in orthopaedic trauma theatres

During the three time periods, the number of hip fracture operations remained similar, ranging from 32-36 procedures per month. However, in April 2020, hip surgeries made up over 40% of all orthopaedic trauma operations, whereas this figure was only 26% the year before (Figure 2).

Operating room efficiency

**Figure 3:**
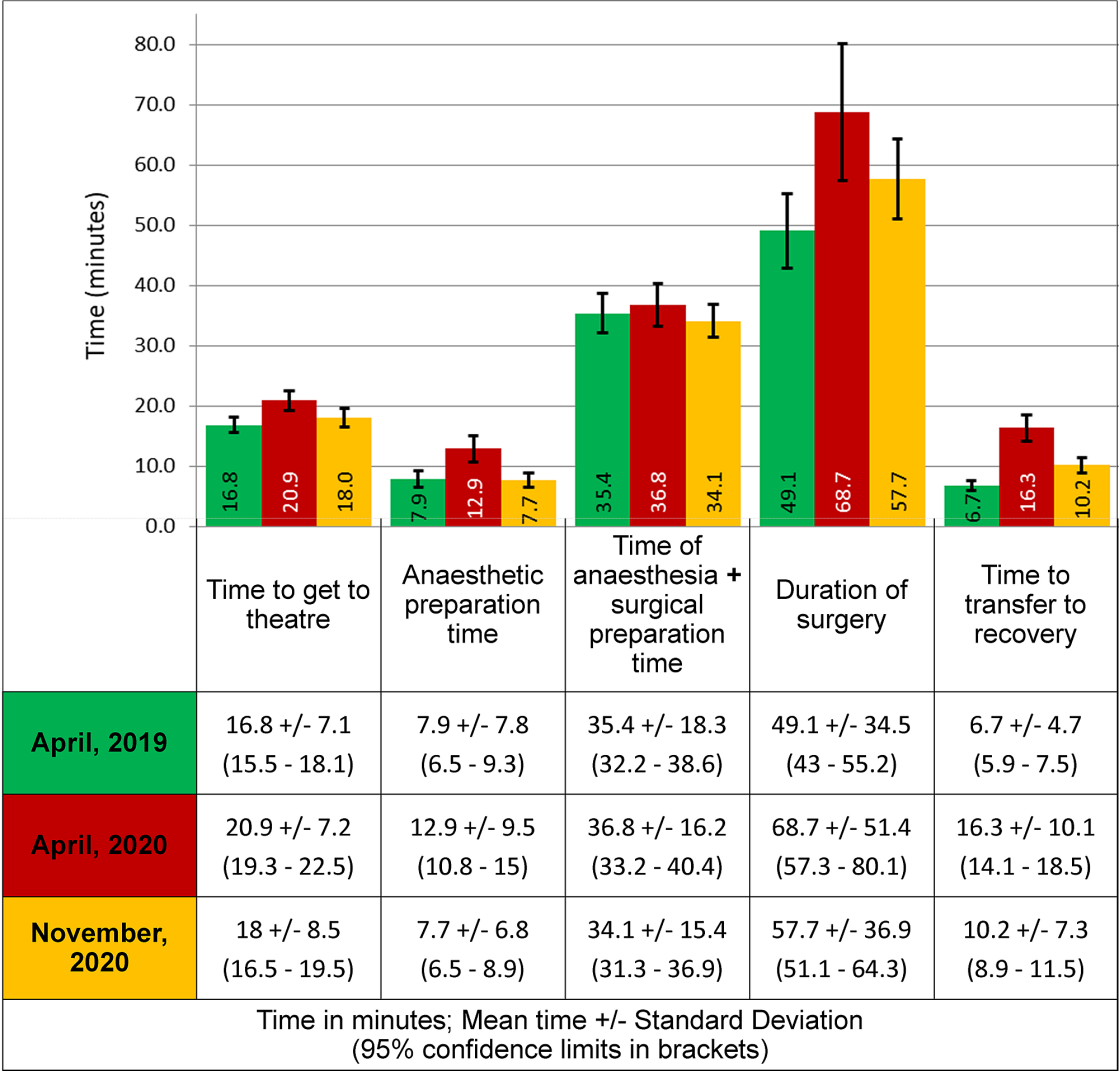
Theatre function times in minutes for the three time periods 95% confidence limits are shown on each of the bars for statistical comparison.

Figure 3 compares theatre function times for the three cohorts of patients. The induction of anaesthesia and surgical preparation time was between 34 and 37 minutes in total in the three time periods with no significant difference (p<0.05). However, all other activities (time to get to the theatre, anaesthetic preparation time, duration of surgery and time to transfer to recovery) took significantly longer during the first wave of COVID-19 pandemic (April 2020) as compared to April 2019, p>0.05).

During the second wave of the pandemic (November 2020), with the exception of anaesthetic and surgical preparation time, all the other activities were performed significantly quicker as compared to the first wave in April 2020 (p<0.05).

When comparisons were made between November 2020 and April 2019, there was no significant difference in the time to get to the theatre, anaesthetic preparation time and the sum of anaesthetic time and surgical preparation time. However, the duration of surgery and time to transfer to recovery was still longer in the second wave compared to April 2019. Table 3 summarises the comparison of the theatre function times for the three cohorts.

**Table 3:** Comparison of theatre function times for the three cohorts

	Time to get to the theatre	Anaesthetic preparation time	Time of anaesthesia + surgical preparation time	Duration of surgery	Time to transfer to recovery
First wave (April 2020) versus Pre-pandemic April 2019	Longer (p=0.000)	Longer (p=0.000)	Same (p=0.296)	Longer (p=0.001)	Longer (p=0.000)
Second wave (November 2020) versus First wave (April 2020)	Shorter (p=0.007)	Shorter (p=0.000)	Same (p=0.123)	Shorter (p=0.040)	Shorter (p=0.000)
Second wave (November 2020) versus Pre-pandemic April 2019	Same (p=0.127)	Same (p=0.432)	Same (p=0.275)	Longer (p=0.031)	Longer (p=0.000)

## Discussion

This study compares cohorts of patients from three time-periods. Weather affects the activity and lifestyle of the general population and seasonal variations in patients presenting to emergency departments across the country is well-documented [13]. However, it is yet uncertain what effect this has on the nature of workload in orthopaedic trauma theatres; the comparison of the cohort of patients in November to that in April may well be influenced by this possible confounding factor. Overall, this study shows a reduction in trauma theatre task efficiency when the pandemic was initially declared, however, results from November 2020 showed an improvement across most measured variables. However, a range of factors needs to be considered while interpreting these results.

The time to get to the theatre and the anaesthetic preparation time both increased during the first wave of the pandemic (April 2020) and then fell again in the second wave (November 2020) to levels which were similar to pre-pandemic times (April 2019). This can be owing to the use of personal protective equipment and social distancing measures in place to control the spread of the virus. However, it is important to note that during the first COVID-19 wave in April 2019, according to hospital policy, all except four patients were anaesthetised in the theatre instead of the anaesthetic room. This was not the case in the second wave of the COVID-19 pandemic where only those patients who were confirmed to have the virus were anaesthetised in theatre. It is, however, difficult to conclude whether this practice had an effect on the changes in the anaesthetic preparation time that were noted.

The results show that there is no significant difference in the sum of time of anaesthesia and surgical preparation time for all three time periods. A similar study by Karia et al. [8], who analysed the time of anaesthesia in isolation for the same time periods (April 2019 and April 2020) also found no significant difference. However, surgical preparation and anaesthesia are both distinct and starkly different functions and one cannot draw any meaningful conclusions while analysing them both grouped together. These functions were not able to be measured in isolation in our department due to the limitations of the data recorded in the electronic database: the time-point when the anaesthetic procedure finished was not recorded, which meant that it was not possible to distinguish between the surgical preparation time from the time of anaesthesia.

The results show that there was a large variation in the duration of surgery for all cohorts. This is because some procedures took less than five minutes in duration, for example, a reduction of a dislocated hip, whereas some involving plate fixation of complex fractures were over four hours in duration. Though the duration of surgeries was shorter in November 2020 as compared to April 2020 (p=0.040), they were still significantly longer as compared to April 2019 (p=0.031). This may be attributed to the change in the type of surgeries that were performed during the COVID-19 pandemic, as noted in Figure 2, with the proportion of hip fracture surgeries being much higher during the pandemic. This practice reflects the guidance of the British Orthopedic Association, which recommends for the treatment of lower limb fragility fractures as a matter of urgency and surgical priority during the pandemic [5]. In addition, due to the lockdown measures in place, the demographics and nature of presentations to hospitals changed, which may have resulted in a lower number of trauma-related injuries requiring surgery at this district general hospital [13-14].

While considering the duration of surgeries, it is also important to note the demographics of patients who underwent operations, as shown in Figure 1. There is an interesting trend observed for patients aged 18-65 who underwent orthopaedic trauma surgery. They made up only 26% of the patients undergoing surgery during the first wave but rose dramatically to 45% during the second wave, which was even greater than the pre-pandemic time-period of April 2019. This is reflected in national trends of patients attending the emergency department (ED) where patients aged 15-44 made less than 3,000 daily attendances to ED in April 2020 and over 5,000 in November 2020 [13].

The time to transfer to recovery was significantly longer in the two waves of the COVID-19 pandemic. It is important to consider the local hospital practices here to understand this. Extubation, which is an aerosol-generating procedure, was performed inside the operating theatres during the first wave for all patients. This may reflect the longer time taken to transfer the patient to recovery during April 2020 where it rose from an average of 6.7 minutes to 16.3 minutes. However, during the second wave, extubation in the theatre was selectively done for patients who were confirmed to have the COVID-19 virus, or for medically unstable patients, which reflects a drop in this time to 10.2 minutes.

Other local hospital practices also need to be taken into account to understand the results. Throughout the pandemic, all COVID-19 infected patients were operated in the same theatre as uninfected patients. This led to a time-consuming ‘deep clean’ of the theatre, which may account for the high number of cancelled cases due to shortage of time (Table 1) and the decreased efficiency of different functions of the theatre (Figure 3) during the pandemic. In addition, the first wave of the pandemic in April 2020 lead to a high number of theatre staff redeployment to medical wards and critical care. This resulted in a significantly reduced, and at times untrained, workforce available to work in orthopaedic trauma theatres. Furthermore, in April 2020, the orthopaedic trauma theatre was moved from its normal location to an operating room in day-case theatres. The staff redeployment and transfer of theatre space, both of which may have resulted in a loss of efficiency, did not occur during the second wave in November 2020.

## Conclusions

The data analysed for the three cohorts shows that the time to get to the theatre, anaesthetic preparation time, duration of surgery and time to transfer to recovery all took significantly longer during the first wave of the COVID-19 pandemic in April 2020. This markedly improved and almost returned to ‘normal’ by November 2020; there is no significant difference in the time to get to theatre and anaesthetic preparation time in November 2020 as compared to the ‘pre-COVID-19’ time period of April 2019. While the duration of surgery and time to transfer to recovery have become shorter during the course of the pandemic, this was still significantly longer as compared to April 2019. In addition, the patient demographics and types of surgeries performed, though initially affected by the first wave of the pandemic, appear to be similar in the second wave to those before the pandemic in April 2019.

Overall, the progression of the pandemic has seen a trend towards a return to ‘normality’ for operating patterns and efficiency in orthopaedic trauma theatres.
